# The value of intraoperative parathyroid hormone monitoring in patients with primary hyperparathyroidism and varying baseline parathyroid hormone levels

**DOI:** 10.1093/bjsopen/zrac118

**Published:** 2022-12-14

**Authors:** Lindsay Hargitai, Carmen Maria Bereuter, Daniela Dunkler, Angelika Geroldinger, Christian Scheuba, Bruno Niederle, Philipp Riss

**Affiliations:** Division of Visceral Surgery, Department of General Surgery, Medical University of Vienna, Vienna, Austria; Division of Visceral Surgery, Department of General Surgery, Medical University of Vienna, Vienna, Austria; Section for Clinical Biometrics, Centre for Medical Statistics, Informatics and Intelligent Systems, Medical University of Vienna, Vienna, Austria; Section for Clinical Biometrics, Centre for Medical Statistics, Informatics and Intelligent Systems, Medical University of Vienna, Vienna, Austria; Division of Visceral Surgery, Department of General Surgery, Medical University of Vienna, Vienna, Austria; Division of Visceral Surgery, Department of General Surgery, Medical University of Vienna, Vienna, Austria; Senior Clinical investigator – Endocrine Surgery, former Chief of the Section of Endocrine Surgery, Medical University of Vienna, Vienna, Austria; Division of Visceral Surgery, Department of General Surgery, Medical University of Vienna, Vienna, Austria

## Abstract

**Background:**

When applying intraoperative parathyroid hormone monitoring (IOPTH) to patients with primary hyperparathyroidism (PHPT), there are established criteria predicting biochemical cure in patients with basal parathyroid hormone (PTH) levels in the medium range (100–400 pg/ml); however, there is a challenge concerning patients with low (less than 100 pg/ml) or high (more than 400 pg/ml) basal PTH levels. The aim of this study was to investigate the value of the ‘Vienna criterion’ applied during IOPTH in patients with PHPT and various basal PTH concentrations.

**Methods:**

Consecutive patients between 1999–2009 with a biochemical diagnosis of PHPT who underwent surgical parathyroidectomy were included. Based on preoperative PTH levels they were divided into three groups: group 1 (low) (<100 pg/ml), group 2 (medium) (100–400 pg/ml) and group 3 (high) (>400 pg/ml) basal PTH. PTH was measured at the start of the operation, when the gland was excised and then at 5, 10 and 15 min after. Calcium and PTH levels were measured at 7 days and 12 months postoperatively. Sensitivity, specificity, positive and negative predictive value, as well as accuracy of IOPTH were calculated for the different groups postoperatively.

**Results:**

675 patients with PHPT were analysed. Sensitivity and specificity were 83.7 per cent and 66.7 per cent in group 1 (*n* = 187), 90.7 per cent and 69.2 per cent in group 2 (*n* = 433), and 94.4 per cent and 100 per cent in group 3 (*n* = 55) to predict cure. Preoperative creatinine (*p* = 0.002) showed significant statistical difference between the groups but was not related to intraoperative PTH decline. At 12 months follow-up normocalcaemia was documented in 98.9 per cent in group 1, 99.0 per cent group 2, and 98.0 per cent of group 3 patients.

**Conclusion:**

Normocalcaemia was predicted intraoperatively by applying the ‘Vienna criterion’ in 98 to 100 per cent and was confirmed after 12 months follow-up in up to 99.0 per cent of patients. Low specificity and a high false-negative rate in patients with low basal PTH show that other criteria might be better suited for this group.

## Teaser

Intraoperative parathyroid hormone (PTH) monitoring (IOPTH) was analysed in 675 patients grouped by basal PTH, and normocalcaemia at 12 months follow-up was observed in 98.8 per cent in group 1 (low), 99.0 per cent group 2 (medium), and 98.1 per cent in group 3 (high). Patients with low basal PTH exhibited low specificity and high false-negative rate, and so other criteria might be better suited for this group. This study confirms that IOPTH is an important tool in parathyroid surgery and without it, would result in a higher rate of disease persistence, thus leading to overall higher costs because of repeated diagnostic procedures and reoperations.

## Introduction

The improvement of preoperative localization procedures and the implementation of intraoperative parathyroid hormone monitoring (IOPTH) has led to an increase of successful minimally invasive, focused (targeted) parathyroid explorations (FNE) through a short skin incision in patients with presumed localized single-gland disease (SGD). This may reduce morbidity with equal success compared with the more extensive bilateral neck exploration (BNE)^1–3^. To exclude multiglandular disease (MGD), even in expected SGD, the application of IOPTH seems mandatory^4^. The American Association of Endocrine Surgeons Guidelines strongly recommends the use of IOPTH to avoid high operating failure rates^5^. The guidelines state that the accuracy of IOPTH depends on the protocol applied, which should be reproducible, practical, and accurate^5^.

As defined by Irvin *et al.*^6^, surgical success was defined as a 50 per cent or more decrease from the highest (pre-incision or pre-excision) value within 10 min after resecting the presumed hyperfunctioning parathyroid tissue. The Miami criterion^7,8^, followed by the Vienna criterion^9^ are documented to be the best-balanced criteria with the highest accuracy in intraoperative prediction of cure compared with the Rome or Halle criteria^10,11^. The Vienna criterion, with its strict definition of basal parathyroid hormone (PTH), improves intraoperative diagnosis of MGD, resulting in an increase in long-term cure by reducing reoperations^9^. ‘Cure’ is intraoperatively predicted if a more than 50 per cent decline from a defined ‘baseline’ PTH level (right after induction of anaesthesia before skin incision) was documented 10 min after removal of the assumed enlarged (hyperfunctioning) gland^9^.

A review of the literature^10–12^ demonstrates that there are various criteria predicting biochemical cure in patients with distinctly (medium) elevated basal PTH levels (100–400 pg/ml); however, its value in patients with mild (less than 100 pg/ml) and very high (more than 400 pg/ml) basal PTH levels is not well established. To date, there is no consensus as to which criteria can help predict cure in these extreme situations. This study aimed to investigate the value of IOPTH applying the Vienna criterion to predict cure in patients with low- or high-elevated compared with medium-elevated basal PTH levels.

## Materials and methods

### Patients

All consecutive patients between 1999 and 2009 with a biochemical diagnosis of primary hyperparathyroidism (PHPT; albumin-adjusted elevated total serum calcium, elevated or inappropriately high normal PTH with high normal or elevated calcium excretion in a 24-h urine sample)^5^ who underwent surgical parathyroidectomy with or without thyroid surgery at an academic hospital were included in this single-centre analysis. Inclusion criteria were sporadic PHPT with available data regarding clinical symptomatology, preoperative ultrasonography and technetium 99m sestamibi (MIBI) scan, method of parathyroidectomy, method of thyroid surgery, final histology, and follow-up data. Exclusion criteria were patients with hereditary disease (multiple endocrine neoplasia; familial hypocalciuric hypercalcaemia; or familial hyperparathyroidism (HPT)), parathyroid carcinoma, ectopic adenoma in the thorax, no preoperative imaging and suspected MGD, or bilateral thyroid nodules requiring initial BNE.

Based on the results of preoperative localization studies, all patients included in this study were scheduled for primary FNE guided by IOPTH and one or more enlarged parathyroid glands were extirpated. In case of thyroid disease documented by ultrasonography with an indication for thyroid surgery, an ipsilateral hemithyroidectomy (lobectomy with isthmus) was performed and patients were included in the study after chart review. The serum creatinine level as a rough estimate of kidney function was included in the preoperative work-up. The glomerular filtration rate representing more precise kidney function was not documented. All patients were treated by the same diagnostic and surgical protocol.

The present study has been approved by the local institutional review board and all patients signed a written informed consent to all diagnostic and surgical procedures.

### Preoperative localization and surgical strategy

Before surgery, all patients had a standard double-phase MIBI scan with single-photon emission CT and high-resolution ultrasonography with colour Doppler of the cervical region to localize one or more enlarged (presumed hyperfunctioning) parathyroid gland(s) and to evaluate the morphology of the thyroid gland (see standard protocols^9,13^). Ultrasonographic examinations were performed by an experienced radiologist and MIBI scans were conducted by an independent nuclear medical physician. All test results were discussed with the surgeon the day before surgery.

Patients with localized SGD on MIBI, irrespective of ultrasonography, underwent FNE (focused, open limited/unilateral exploration with selective extirpation of parathyroid glands(s); skin incision 20–25 mm^13^). If a MIBI scan was negative, FNE was started on the side showing positive ultrasonography^13^. Patients with both negative MIBI scans and ultrasonography, and/or contralateral or bilateral thyroid disease, underwent BNE^13^. In patients with additional thyroid surgery, a hemithyroidectomy was performed after removing the enlarged parathyroid glands and after interpretation of IOPTH. The results of additional thyroid surgery are not the subject of this analysis and have been presented and discussed extensively in previous studies^14^.

### PTH measurements and IOPTH

A commercially available two-step sandwich electrochemiluminescence intact PTH immunoassay (Roche® Germany) run on the Elecsys 1010© Roche (Germany) Test System was used in all patients to determine PTH levels before surgery and to monitor PTH course during surgery. Peripheral blood samples were taken at the beginning of the operation (baseline), at the time of extirpation of the enlarged gland(s), and 5, 10 and 15 min after.

### Surgical strategy

If the enlarged (hyperfunctioning) gland could not be localized at the presumed location during FNE (or if IOPTH decrease was inadequate; see below), the focused exploration was extended to a BNE to localize the single gland in another location or to uncover multiple gland disease to avoid surgical failure (persisting disease).

If additional thyroid surgery was performed, PTH decline was always documented before thyroid surgery and after ‘adequate’ parathyroid surgery.

### PTH groups

Based on the preoperative basal PTH values, the patients were divided into three different groups: group 1, low basal PTH (less than 100 pg/ml); group 2, medium basal PTH (100–400 pg/ml); and group 3, high basal PTH (more than 400 pg/ml). Group 1 was chosen based on results^15^ demonstrating a significantly higher rate of MGD in patients with ‘low’ basal PTH (less than 100 pg/ml). In addition, these patients displayed differences in PTH kinetics compared with patients with ‘high’ basal PTH. Group 3 was chosen based on a publication that states that the PTH levels should fall within the normal range after 15 min^16^. The hypothesis was that patients with ‘high’ basal PTH (more than 400 pg/ml) may reach the 50 per cent cut-off after 10 min, but not fall within the normal range, which may indicate a persisting increased parathyroid metabolism.

### Follow-up

Both calcium and PTH were measured at 7 days to confirm the intraoperative prediction of postoperative parathyroid metabolism (‘cure’), and also at the 12-month follow-up.

‘Cured’ patients were those whose calcium and PTH values were within the normal range (calcium of 2.20–2.55 mmol/l and PTH of 15–65 ng/l) during follow-up. A recent publication^17^ has identified a small group of ‘cured’ patients with short, temporary ‘mild’ hypocalcaemia (calcium less than 2.20 mmol/l and PTH less than 15 ng/l) within the first 7 days after surgery. These patients are mostly asymptomatic and only a minority of them require calcium and vitamin D3 supplementation to maintain calcium in the normal range and to treat the mild hypocalcaemic symptoms. In long-term follow-up these patients are by definition ‘cured’.

‘Persisting disease’ was defined as calcium and PTH still elevated at 6 months after surgery. Patients in whom calcium and PTH values were initially documented in the normal range for at least 6 months and in whom an increase in calcium and PTH levels was later observed were defined as having ‘disease recurrence’. Permanent hypocalcaemic patients exhibited calcium less than 2.20 mmol/l and PTH less than 15 pg/ml as well as hypocalcaemic symptoms necessitating calcium and vitamin D3 supplementation to maintain calcium in the normal range (2.20 mmol/l or higher) at 12-month follow-up.

At the 12-month follow-up, patients were re-evaluated to document the number of normocalcaemic, (persisting/recurring) hypercalcaemic and permanent hypocalcaemic patients in each subgroup.

### Statistical analysis

Categorical variables are reported as absolute numbers and percentages, whereas continuous variables are given as median and first and third quartiles. Fisher's exact test and Kruskal–Wallis test was used to compare categorical and continuous variables between groups respectively. To assess correlation between continuous variables Spearman correlation coefficient was used.

The correctness of the intraoperative interpretation of PTH decline when applying the Vienna Criterion, which influenced the extent of exploration, was verified based on the biochemical parameters seven days postoperatively.

True positives (TPs) were defined as correct intraoperative prediction of permanent normocalcaemia (‘cure’). Patients, where the PTH decline was less than 50 per cent and therefore a more extended exploration was mandatory to avoid surgical failure (persisting disease) were classified as true negatives (TNs). The more extended exploration should lead to cure. False negatives (FNs) were defined as incorrect predictions of persisting disease, which were an insufficient decline of PTH but where normocalcaemia was documented. False positives (FPs) were defined as incorrect predictions of complete resection, resulting in persistent hypercalcaemia.

Sensitivity, specificity, positive predictive value (PPV), negative predicative value (NPV), and accuracy were calculated within and across the three PTH groups. Exact 95 per cent confidence intervals (c.i.) were calculated. The SAS system version 9.4 (SAS Institute, Cary, NC, USA) was used for statistical calculations^18^.

## Results

### Patients

During the study interval, 1021 patients with the biochemical diagnosis of PHPT were treated surgically. Overall, 675 patients (518 female, 76.7 per cent) with sporadic PHPT on whom prospectively collected data regarding clinical symptomatology, presumed localized SGD by preoperative imaging, characteristic preoperative biochemical parameters, extent of surgery, date of surgery, final histology, and follow-up were available were included in the retrospective analysis. Patient characteristics are described in detail in *Table 1*.

**Table 1:** Patient demographics, characteristics, and surgical procedures

		Group 1 (low)	Group 2 (medium)	Group 3 (high)	Total	*P*
		<100 pg/ml PTH	100–400 pg/ml PTH	>400 pg/ml PTH		
	* *	187 (27.7)	433 (64.2)	55 (8.1)	675	
**Sex ratio (M:F)**		36 (19.3):151 (80.8)	100 (23.1):333 (76.9)	21 (38.2):34 (61.8)	157 (23.3):518 (76.7)	0.017
**Biochemistry**						
Preoperative PTH (pg/ml) median (5th and 95th percentile)	Normal range 15–65	77.3 (41.9, 98.0)	165.0 (104.2, 340.0)	581.0 (416.0, 1521.0)	138.0 (57.0, 498.0)	
Calcium (mmol/l) median (5th and 95th percentile)	Normal range 2.20–2.55	2.73 (2.52, 3.17)	2.80 (2.52, 3.22)	2.88 (2.53, 3.43)	2.78 (2.52, 3.25)	0.001
Creatinine (mg/dl) median (5th and 95th percentile)	Normal range <1.3	0.90 (0.68, 1.22)	0.87 (0.65, 1.47)	0.98 (0.62, 1.99)	0.89 (0.67, 1.47)	0.002
**Morphology**	SGD	180 (96.3)	416 (96.1)	51 (92.7)	647 (95.9)	0.452
	MGD	7 (3.7)	17 (3.9)	4 (7.2)	28 (4.1)	
**Surgery**	FNE	167 (89.3)	387 (89.4)	49 (89.1)	603 (89.3)	0.979
	Conversion to BNE	20 (10.7)	46 (10.6)	6 (10.9)	72 (10.7)	

Values are *n* (%) unless otherwise indicated. Fisher's exact test and Kruskal–Wallis test were used to compare categorical and continuous variables between group respectively. PTH, parathyroid hormone; FNE, open minimally invasive targeted/selected and unilateral exploration with extirpation of one enlarged parathyroid gland; BNE, bilateral neck exploration; SGD, single-gland disease; MGD, multiple gland disease.

### Surgical procedures

Based on preoperative imaging and IOPTH monitoring, a total of 603 of 675 (89.3 per cent) patients underwent successful FNE. In 72 of 675 (10.7 per cent) FNE was converted to BNE due to an inconclusive IOPTH decline based on a false localization or unexpected MGD. In total, 176 (26.1 per cent) patients underwent an additional hemithyroidectomy due to unilateral thyroid lesions.

### Study groups based on basal PTH values

Of the 675 patients, 187 (27.7 per cent) were group 1 (low), 433 (64.2 per cent) were group 2 (medium), and 55 (8.1 per cent) were group 3 (high) (*Table 1*). The median relative intraoperative PTH decline in group 1, 2, and 3 was 70.9 per cent, 77.8 per cent and 85.0 per cent respectively.

### Preoperative creatinine and baseline PTH

Preoperative calcium (*P* = 0.001) and preoperative creatinine levels (*P* = 0.002) were significantly different between the three groups (*Table 1*); however, no correlation was seen between preoperative creatinine and intraoperative PTH decline after 10 min, regardless of the group (Spearman correlation 0.049, *P* = 0.204).

### Parathyroid morphology

Overall, 647 (95.9 per cent) patients had SGD and 28 (4.1 per cent) had MGD. SGD was documented in 180 (96.3 per cent) group 1 patients; 416 (96.1 per cent) group 2 patients; and 51 (92.7 per cent) group 3 patients (*Table 1*). In total, 23 (82.1 per cent) patients underwent subtotal parathyroidectomy for MGD and five (17.9 per cent) patients had two enlarged glands classified as ‘double adenomas’ removed.

### IOPTH decline 10 min after excision of the presumed hyperfunctioning parathyroid gland and accuracy of Vienna criterion predicting cure

The sensitivity, specificity, PPV, NPV, and accuracy when applying the Vienna criterion to IOPTH decline are shown in *Table 2*. Sensitivity, specificity, and overall accuracy increased along the groups, with values of 83.7 per cent, 66.7 per cent and 82.9 per cent for group 1, 90.7 per cent, 69.2 per cent, and 89.4 per cent for group 2, and 94.4 per cent, 100 per cent, and 94.6 per cent for group 3.

**Table 2:** Accuracy of the Vienna criterion (confirmation 7 days after surgery) for each group

	Group 1 (low)	Group 2 (medium)	Group 3 (high)	All
	PTH <100 pg/ml	PTH 100–400 pg/ml	PTH >400 pg/ml	
	*n* = 187	*n* = 433	*n* = 55	
Sensitivity	83.7 (77.5, 88.8)	90.7 (87.4, 93.3)	94.4 (84.6, 98.8)	89.1 (86.4, 91.4)
Specificity	66.7 (29.9, 92.5)	69.2 (48.2, 85.7)	100 (2.5, 100)	69.4 (51.9, 83.7)
Accuracy	82.9 (76.7, 88.0)	89.4 (86.1 92.1)	94.6 (84.9, 98.9)	88.0 (85.3, 90.4)
PPV	98.0 (94.3, 99.6)	97.9 (95.9, 99.1)	100 (93.0, 100)	98.1 (96.6, 99.1)
NPV	17.1 (6.6, 33.7)	32.1 (20.3, 46.0)	25.0 (0.6, 80.6)	26.3 (17.8, 36.4)

Values are accuracy (95 per cent c.i.). NPV, negative predictive value; PPV, positive predictive value; PTH, parathyroid hormone.

### Biochemical follow-up at 7 days after surgery to confirm IOPTH monitoring

In group 1, 165 (88.2 per cent) patients were normocalcaemic 7 days after surgery and 19 (10.2 per cent) patients had mild hypocalcaemia (18 patients received intermittent postoperative therapy with calcium and vitamin D and 1 patient received calcium substitution intravenously). Three (1.6 per cent) patients were persistently hypercalcaemic.

In group 2, 372 (85.9 per cent) patients were normocalcaemic, 54 (12.5 per cent) had mild hypocalcaemia (all received intermittent postoperative therapy with calcium and vitamin D orally) and seven (1.6 per cent) patients were hypercalcaemic.

In group 3, 35 (63.6 per cent) patients were normocalcaemic and 20 (36.4 per cent) patients had mild hypocalcaemia (all patients received intermittent postoperative therapy with calcium and vitamin D; *Tables 3–5*).

**Table 3:** Group 1: preoperative ‘low’ parathyroid hormone levels. Intraoperative interpretation of the parathyroid hormone decline (Vienna criterion; including exact 95 per cent confidence intervals), 7-day and 12-month biochemical follow-up results

Group 1 (low) *n* = 187					
	TP 149 (79.7)	TN 6 (3.2)	FN 29 (15.5)	FP 3 (1.6)	Total ∑*n* (%)
**Surgery**					
FNE	139 (93.3)	0	25 (86.2)	3 (100)	167 (89.3)
Conversion to BNE	10 (6.7)	6 (100)	4 (13.8)	0	20 (10.7)
**Morphology**					
SGD	149 (100)	0	29 (100)	2 (66.7)	180 (96.3)
MGD	0	6 (100)	0	1 (33.3)	7 (3.7)
					187 (100)
**7-day follow-up**					
**Biochemically cured**					
Normocalcaemia†	136 (91.3)	4 (66.7)	25 (86.2)	0	165 (88.2)
Mild hypocalcaemia‡	13 (8.7)	2 (33.3)	4 (13.8)	0	19 (10.2)
**Biochemically not cured**					
Hypercalcaemia/persistence§	0	0	0	3 (100)	3 (1.6)
Hypocalcaemia¶	0	0	0	0	0
Reoperation	0	0	0	2 (66.7)	2 (1.1)
**12-month follow-up**					
Lost to follow-up	5 (3.4)	0	1 (3.4)	0	6 (3.2)
**Biochemically cured**					
Normocalcaemia†	143 (99.3)	6 (100)	28 (96.6)	2 (66.7)	179 (98.8)
Hypercalcaemia/persistence	0	0	0	1# (33.3)	1 (0.6)
Hypocalcaemia¶	1 (0.7)	0	0	0	1 (0.6)

Values are *n* (%). d, days; mo, months; TP, true positive; TN, true negative; FN, false negative; FP, false positive; FNE, focused neck exploration; BNE, bilateral neck exploration; SGD, single-gland disease; PTH, parathyroid hormone; MGD, multi-gland disease >2 enlarged glands removed.

*Conversion FNE.

†Normocalcaemia: normal parathyroid function: calcium ≥ 2.20 and ≤2.55 mmol/l, PTH ≥ 15 and ≤65 ng/l.

‡Mild hypocalcaemia: calcium < 2.20 mmol/l, PTH < 15 ng/l (formal definition). Calcium and vitamin D3 supplementation was necessary to maintain calcium in the normal range (≥2.20 mmol/l) in symptomatic patients and in those with PTH < 10 ng/l.

§Hypercalcaemia with persisting disease: calcium > 2.55 mmol/l, PTH > 65 ng/l.

¶Hypocalcaemia with permanent hypoparathyroidism: calcium < 2.20 mmol/l and PTH < 15 pg/ml and hypocalcaemic symptoms necessitating calcium and vitamin D3 supplementation.

#No reoperation.

**Table 4:** Group 2: preoperative ‘medium’ parathyroid hormone levels. Intraoperative interpretation of the parathyroid hormone decline (Vienna criterion; including exact 95 per cent confidence intervals), 7-day and 12-month biochemical follow-up results

Group 2 (medium) *n* = 433					
							TP 369 (85.3)	TN 18 (4.2)	FN 38 (8.8)	FP	Total ∑*n* (%)
**Surgery**					
FNE	340 (92.1)	4 (22.2)	35 (92.1)	8 (100)	387 (89.4)
Conversion to BNE	29 (7.9)	14 (77.8)	3 (7.9)	0	46 (10.6)
**Morphology**					
SGD	368 (99.7)	3 (16.7)	38 (100)	7 (87.5)	416 (96.1)
MGD	1 (0.3)	15 (83.3)	0	1 (12.5)	17 (3.9)
					433 (100)
**7-day follow-up**					
**Biochemically cured**					
Normocalcaemia†	328 (88.9)	11 (61.1)	32 (84.2)	1 (12.5)	372 (85.9)
Mild hypocalcaemia‡	41 (11.1)	6 (33.3)	6 (15.8)	1 (12.5)	54 (12.5)
**Biochemically not cured**					
Hypercalcaemia/persistence§	0	1 (5.6)	0	6 (75.0)	7 (1.6)
Hypocalcaemia¶	0	0	0	0	0
Reoperation	0	0	0	5 (62.5)	5 (1.2)
**12-month follow-up**					
Lost to follow-up	22 (6.0)	1 (5.6)	1 (2.6)	0	24 (5.5)
**Biochemistry**					
Normocalcaemia†	347 (100)	16 (94.1)	37 (100)	5 (62.5)	405 (99.0)
Hypercalcaemia/persistence§	0	1 (5.9)	0	3 (37.5)	4 (1.0)
Hypocalcaemia¶	0	0	0	0	0

Values are *n* (%). d, days; mo, months; TP, true positive; TN, true negative; FN, false negative; FP, false positive; FNE, focused neck exploration; BNE, bilateral neck exploration; SGD, single-gland disease; MGD, multi-gland disease >2 enlarged glands removed.

* Conversion FNE.

†Normocalcaemia: normal parathyroid function: calcium ≥ 2.20 and ≤2.55 mmol/l, PTH ≥ 15 and ≤65 ng/l.

‡Mild hypocalcaemia: calcium < 2.20 mmol/l, PTH < 15 ng/l (formal definition) and where calcium and vitamin D3 supplementation was necessary to maintain calcium in the normal range (≥2.20 mmol/l) in symptomatic patients and in those with PTH < 10 ng/l.

§Hypercalcaemia with persisting disease: calcium > 2.55 mmol/l and PTH > 65 ng/l.

¶Hypocalcaemia with permanent hypoparathyroidism: calcium < 2.20 mmol/l and PTH < 15 pg/ml and hypocalcaemic symptoms necessitating calcium and vitamin D3 supplementation.

**Table 5:** Group 3: preoperative ‘high’ parathyroid hormone levels. Intraoperative interpretation of the parathyroid hormone decline (Vienna criterion, including exact 95 per cent confidence intervals), 7-day and 12-month biochemical follow-up results

Group 3 (high) *n* = 55					
							TP 51 (92.7)	TN 1 (1.8)	FN 3 (5.5)	FP 0	Total ∑*n* (%)
**Surgery**					
FNE	46 (90.2)	0	3 (100)	0	49 (89.1)
Conversion to BNE	5 (9.8)	1 (100)	0	0	6 (10.9)
**Morphology**					
SGD	48 (94.1)	0	3 (100)	0	51 (92.7)
MGD	3 (5.9)	1 (100)	0	0	4 (7.3)
					55 (100)
**7-day follow-up**					
**Biochemically cured**					
Normocalcaemia†	32 (62.7)	0	3 (100)	0	35 (63.6)
Mild hypocalcaemia‡	19 (37.3)	1 (100)	0	0	20 (36.4)
**Biochemically not cured**					
Hypercalcaemia/persistence§	0	0	0	0	0
Hypocalcaemia¶	0	0	0	0	0
Reoperation	0	0	0	0	0
**12-month follow-up**					
Lost to follow-up	3 (5.9)	0	0	0	3 (5.5)
**Biochemistry**					
Normocalcaemia†	48 (100)	0	3 (100)	0	51 (98.1)
Hypercalcaemia/persistence§	0	0	0	0	0
Hypocalcaemia¶	0	1 (100)	0	0	1 (1.9)

Values are *n* (%). d, days; mo, months; TP, true positive; TN, true negative; FN, false negative; FP, false positive; FNE, focused neck exploration; BNE, bilateral neck exploration; SGD, single-gland disease; PTH, parathyroid hormone; MGD, multi-gland disease >2 enlarged glands removed.

* Conversion FNE.

†Normocalcaemia: normal parathyroid function, calcium ≥ 2.20 and ≤2.55 mmol/l, PTH ≥ 15 and ≤65 ng/l.

‡Mild hypocalcaemia: calcium < 2.20 mmol/l, PTH < 15 ng/l (formal definition) and where calcium and vitamin D3 supplementation was necessary to maintain calcium in the normal range (≥2.20 mmol/l) in symptomatic patients and in those with PTH < 10 ng/l.

§Hypercalcaemia with persisting disease: calcium > 2.55 mmol/l and PTH > 65 ng/l.

¶Hypocalcaemia with permanent hypoparathyroidism: calcium < 2.20 mmol/l and PTH < 15 pg/ml and hypocalcaemic symptoms necessitating calcium and vitamin D3 supplementation.

### Biochemical follow-up 12 months after surgery

In group 1, 179 (98.9 per cent) patients were normocalcaemic without calcium or vitamin D substitution at 12 months and were, by definition, cured. This includes two out of three patients with persisting HPT after 7 days who underwent successful reoperation within 12 months. In one (0.6 per cent) patient with persisting hypercalcaemia, the hyperfunctioning parathyroid gland could not be localized and a further one (0.6 per cent) patient was still hypocalcaemic following initial BNE with subtotal parathyroidectomy for MGD, thymectomy, and hemithyroidectomy for unilateral thyroid nodules with PTH in the low-normal range. No recurrences were observed.

In group 2, of the seven patients presented with persisting disease at 7 days, five patients underwent reoperation and two patients (one preliminarily classified as TN and one as FP) did not, as the hyperfunctioning tumours could not be located. Reoperation was successful in three out of five patients. Despite reoperation, two patients continued to have persistent hypercalcaemia. Therefore, a total of four (1.0 per cent) patients showed persistent hypercalcaemia (one patient was marginally persistent despite removal of two parathyroids and additional work-up revealed no genetic causes nor other hyperfunctioning parathyroids; in three others no hyperfunctioning parathyroids could be localized with high probability). No hypocalcaemia or recurrence was seen. After 12 months, the overall cure rate of group 2 patients (including the successful reoperations) was 405 of 409 patients (99.0 per cent).

In group 3, 51 of 52 (98.1 per cent) patients were normocalcaemic and one (1.9 per cent) patient was hypocalcaemic following BNE, hemithyroidectomy, and additional prolonged increased bone metabolism because of pronounced osteoporosis. At the 12-month follow-up the patient demonstrated hypocalcaemia substituted with oral calcium, but had normal PTH levels. No persistence or recurrence was observed.

## Discussion

According to a study by Harrison *et al.*^11^, clear recommendations on the use of IOPTH during surgery for PHPT is demonstrated in the literature. IOPTH is an important tool in parathyroid surgery and recent studies have demonstrated its use in at least 74 per cent of PHPT operations^19^. The present study investigated 675 consecutive patients with biochemically confirmed PHPT and presumed SGD by preoperative imaging by applying IOPTH and the Vienna criterion in patients retrospectively grouped corresponding to low (less than 100 pg/ml), medium (100–400 pg/ml), and high (more than 400 pg/ml) basal PTH. All procedures followed a prospective protocol, allowing for retrospective analysis of a homogenous patient population.

When applying the Vienna criterion, ‘cure’ was positively predicted in 98 per cent in group 1, 97.9 per cent in group 2, and 100 per cent in group 3 respectively; however, 7 days after surgery, normocalcaemia was only documented in 88.2 per cent of group 1, 85.9 per cent of group 2 and 63.6 per cent of group 3 patients respectively. At that time ‘mild’ hypocalcaemia was observed in 10.2 per cent, 12.5 per cent and 36.4 per cent in groups 1, 2, and 3 respectively. In the literature, normocalcaemia is typically assessed within the first week after surgery; however, several studies have demonstrated that bone remodelling is still in the initial phase at this time and has not yet been completed 7 days after surgery^17,20^. As shown in group 3, a more pronounced bone turnover may be suspected, resulting in a greater number of ‘mildly’ hypocalcaemic patients requiring calcium and vitamin D substitution^17^. Kaderli *et al.*^17,20^ recommended supplementation with calcium and vitamin D routinely to improve bone metabolism efficiently. Therefore, this study analysed the surgical success by evaluating the parathyroid metabolism after 12 months, which seems more representative for cure.

Permanent hypocalcaemia occurs more often following BNE^17,20^, being documented in 0.5–3.8 per cent^21^. In this study, 2 of 641 (0.3 per cent) patients had hypocalcaemia 12 months following BNE. The number of parathyroids removed correlates well with (long-term) hypocalcaemia^1,22^.

After reviewing long-term follow-up, persisting hypercalcaemia is reported in the literature in 2.5–5 per cent of sporadic PTH^23^. Persisting hypercalcaemia was revealed in 5 of 641 (0.8 per cent) patients, which is lower than in the literature. Only one patient (0.6 per cent) demonstrated persistence in group 1, four patients (1.0 per cent) in group 2 and none in group 3, which also reflects in the low conversion rate from FNE to BNE in all three groups. According to a study by Yeh *et al.*^23^, predictive factors for persistence may include age more than 70 years, obesity, low hospital volume, low surgeon experience, equivocal MIBI scan results, initial parathyroid pathology (single adenoma < double adenoma < multiglandular disease) and surgical strategy. The low persistence rate observed in this analysis may be due to the fact, that patients were treated by highly experienced endocrine surgeons.

Preoperative calcium and creatinine showed significant statistical differences between the three groups. This can be expected, considering in vivo studies demonstrating that the PTH–calcium curve is shifted to the right and the set point for calcium is increased in patients with PHPT^24^. Essentially, a high PTH value is a response to a high calcium concentration, given that the PTH–calcium relationship is bifunctional; calcium concentration controls PTH secretion and at the same time, PTH regulates calcium concentration^25^. Furthermore, a decline in renal function due to hypercalcaemia decreases phosphorus excretion, which compromises the calcaemic action of PTH due to a loss of parathyroid gland sensitivity to the control by serum concentrations of calcium and phosphorus due to an increase in serum phosphorus^26–28^. As expected, higher PTH values were associated with higher preoperative calcium and creatinine values in this study.

Overall, 28 (4.1 per cent) patients had MGD, 23 patients underwent subtotal parathyroidectomy, and five patients had two enlarged glands removed. In the literature, the number of patients with ‘low’ basal PTH and MGD lies between 26–60 per cent^15,29–33^; however, in this study, only 3.7 per cent with MGD had ‘low’ basal PTH. A similar rate of 3.9 per cent was observed in group 2. In addition, the number of patients with MGD and ‘high’ baseline PTH in the literature lies between 10–15 per cent^34,35^. Interestingly, this study only observed 7.2 per cent; however, the finding that this rate is higher than in the other two groups is likely to be a random finding that is attributed to the small number of patients in this group. The rate of MGD did not differ significantly between the three groups. Several studies have shown that stricter criteria for MGD patients need to be defined, given that parameters such as size and weight are not reliable predictors of disease as some enlarged glands may not be hyperfunctioning^36,37^. As observed by Riss *et al.*^38^, not only is a high specificity required to not oversee patients with MGD, strict definition of a PTH ‘baseline’ improves intraoperative diagnosis of MGD, thus reducing the extension of surgery (and indirectly morbidity), reoperations, and increasing long-term cure.

The present study demonstrates that the intraoperative application of the Vienna criterion could precisely predict long-term cure independent of the basal PTH level in between 98 and 99 per cent of patients. Sensitivity and specificity are all highest in group 3 and lowest in group 1. Thus, the lowest accuracy is observed in group 1 and the highest in group 3. The interpretation of the PTH decline was more complex in group 1 starting with a ‘low’ basal PTH. To date, there is an unclear definition of ‘more than 50 per cent decline’ of PTH 10 min after removal of the presumed hyperfunctioning gland, thus resulting in a higher number of FN findings (15.5 per cent) compared with the two other PTH groups (8.8 per cent and 5.5 per cent). After excision, a postulated decrease of 50 per cent or more from baseline value could result in PTH already falling within the normal range, but the patient is not yet cured. Therefore, this subgroup of FN patients in group 1 are the most problematic in terms of predicting cure rates. In this study, a conversion to BNE was more often undertaken compared with the two other groups without localizing a second adenoma or MGD in patients with low basal PTH.

When analysing the IOPTH results, one must also take into consideration any influence that may have occurred during the operation, such as ‘PTH spikes’ through manipulation of the gland that can lead to higher PTH values^39^. During exploration an ‘intended intraoperative manipulation’ of parathyroid adenomas through mechanical stimulation may lead to increased PTH excretion^40,41^. According to Riss *et al.*^42,43^, PTH spikes may be caused by intraoperative manipulation of the enlarged gland during preparation in around 15 per cent, leading to a slower PTH decay. This manipulation may also increase PTH levels to very high levels and therefore may influence the intraoperative interpretation of the PTH decrease. In this situation, the PTH decline must be assessed individually on a per-patient basis instead of strictly adhering to a specific criterion^40^. The application of the Miami criterion seems more accurate after gland manipulation^40,44^. Even waiting for 15 or 20-min post-excision values if IOPTH decline is not adequate in patients in group 3 is sometimes helpful to predict cure instead of extending the exploration.

Another source of influence in IOPTH monitoring is renal insufficiency, which alters the half-life of PTH^9,45^. Studies have shown that kidney function, measured by preoperative creatinine, may have an influence on intraoperative PTH decline because of cross-reactions with PTH metabolites^46,47^; however, several studies have shown that patients with chronic kidney disease that undergo parathyroidectomy demonstrate IOPTH degradation kinetics similar to patients with normal kidney function, thus, the same IOPTH decline should be utilized in patients with normal kidney function and chronic kidney disease^48,49^. In this study, no correlation was found between preoperative creatinine and intraoperative PTH decline within the various groups, which is similar to the literature^50^. These results also strengthen the recommendation that the same IOPTH decline should be used in patients with normal and chronic kidney function.

There are some limitations in this study protocol. Although all parameters were collected prospectively, the interpretation of the results was performed retrospectively. Gathering more experience with time in selected patients (for example manipulated PTH values), the interpretation of the PTH decline has to be individualized. Therefore, this must be considered in a redesigned prospective study. The study has an overall large population size, but the group sizes were very different, leading to difficulties in identifying statistically significant relationships in the data and between the groups.

Dependent on the three basal PTH groups, normocalcaemia was predicted intraoperatively by applying the Vienna criterion in 98–100 per cent and was confirmed after the 12-month follow-up in up to 99.0 per cent of patients. While preoperative creatinine showed a significant statistical difference between the three groups, it did not influence IOPTH. Low specificity and a high FN rate of IOPTH in patients with ‘low’ basal PTH shows that other criteria might be better suited for this group of patients. This study confirms that IOPTH is still an important tool in parathyroid surgery and not measuring it would more than double the rate of disease persistence^4^, thus leading to overall higher costs^4^ as a result of repeated diagnostic procedures and reoperations.

## Data Availability

The data sets generated and/or analysed during the present study are available from the corresponding author on reasonable request.
